# An Ego Depletion Perspective Linking Political Behavior to Interpersonal Deviance

**DOI:** 10.3389/fpsyg.2022.802636

**Published:** 2022-03-30

**Authors:** Jing Xiu, Junwei Zheng, Zhigang Li, Zhenduo Zhang

**Affiliations:** ^1^School of Applied Economics, University of Chinese Academy of Social Sciences, Beijing, China; ^2^Faculty of Civil Engineering and Mechanics, Kunming University of Science and Technology, Kunming, China; ^3^School of Economics and Management, Beijing Polytechnic, Beijing, China; ^4^School of Economics and Management, Dalian University of Technology, Dalian, China

**Keywords:** political acts, interpersonal deviance, moral self-efficacy, chronic job strain, experience sampling method

## Abstract

A political act is a typical self-serving behavior that works to promote or protect self-interest. However, limited research explores its relationship with daily downstream behavior. Based on the ego depletion theory, the present study attempts to clarify when and how daily political acts will be transformed into interpersonal deviance. We collected 760 cases nested in 152 full time workers in mainland China through the experience sampling method. *Via* a multilevel structural equation model and hierarchical linear model, we tested the mediated moderation model. The results showed that political acts correlated with interpersonal deviance on a daily basis. Moral self-efficacy buffers the relationship between political acts and interpersonal deviance, whereas chronic job strain amplifies the relationship. Furthermore, moral self-efficacy can mediate the moderating role of chronic job strain.

## Introduction

Political acts are defined as informal and discretionary activities aimed at promoting or protecting self-interest by influencing the thinking, perception, or behavior of other members of the organization (Perrewé et al., 2012; Gabriel et al., 2018). In contrast to helping behavior, the political act is pro-self and not formally endorsed or supported by organizations (Gabriel et al., 2018). Although prior literature is theoretically addressing the detrimental effects of political acts, studies are predominantly focusing on the antecedents of political acts (Hochwarter et al., 2003; Gabriel et al., 2018). The effects of performing political acts on actors’ downstream behaviors and well-being have mostly been overlooked. Exploring such effects is important because knowledge of the potential costs and benefits of political acts for actors can be leveraged to aid political acts management, which is attracting increased attention from practitioners and management researchers (Chang et al., 2009). For instance, understanding why political acts cause harmful consequences might be used to develop coping strategies in order to inhibit the unexpected outcomes.

To address these questions, we examine the possible unfavorable impacts of engaging in political act for employees from an actor-centric perspective. We direct our attention to temporal or proximal consequences of political acts, as several empirical studies have shown that political acts fluctuate on a daily basis (Gabriel et al., 2018). To better understand these fluctuations, we draw upon the ego depletion theory (Baumeister et al., 1998), as it provides particularly relevant and helpful insight into the more immediate actor-centric consequences of daily behaviors (Trougakos et al., 2015). The core tenet of the ego depletion theory is that self-control is a limited resource, which is used to suppress impulses and regulate personal behavior, and to sustain executive functioning for the purpose of achieving preset goals (Barlett et al., 2016). Whenever people engage in activities requiring high degrees of self-control, attentional resources are consumed, thus diminishing their capacity for self-control on regulating downstream behaviors and quelling unethical urges [e.g., ridiculing or being to others (Eissa et al., 2019)].

Time at work is limited because workdays are segmented into a finite number of performance episodes organized around relevant work goals (Koopman et al., 2016). Employees are generally driven to make progress toward work goals (Han et al., 2016), but political acts compete for time at the expense of that progress. Due to this, political acts were viewed as a special kind of organizational deviance which violated task performance (Breaux et al., 2009; Sun and Chen, 2017). The impeded progress may in turn prompt employees to direct more attention resources to their work and may lead to consistent depletion of self-control resources and energy (Lin et al., 2017). Employees in ego depleted states are more likely to engage in interpersonal deviance for the short-lived benefits in order to recover from the ego depletion (Ruci et al., 2018). Moreover, political acts were instrumental aiming to exert influences on coworkers which caused changes in interpersonal relationships (Cheng et al., 2022). Interpersonal deviance was sensitive to such changes and was adopted to retain the control in interpersonal interactions (Wu et al., 2014). Based on and extending prior research, *the first research question of the present study is whether daily political acts are positively associated with interpersonal deviance.*

Moreover, literature on ego depletion theory posits that personal characteristics shape individual’s ego depletion processes (Rosen et al., 2016; Chen et al., 2018). Activities that create additional stress for actors deplete more additional self-control resources (Prem et al., 2016). Job strain and moral self-efficacy are two critical personal characteristics which shape individuals’ psychological and behavioral responses when they are in ego depleted states (Chen et al., 2018; Zhang et al., 2021). Accordingly, we extend our research by identifying individual factors, such as job strain and moral self-efficacy, which moderate the costs of political acts since they determine whether political acts cause additional stress in the present study.

Job strain is a subjective reaction in response to an unpleasant experience evoked by particularly stressful work events or conditions (Hurrell et al., 1998; Schmitt et al., 2016). When an individual feels strained, mobilization effort and resources are required to ensure effective functioning (Hurrell et al., 1998; Schmitt et al., 2016). Thus, the resource depletion process will be stronger under the condition of high job strain. Moral self-efficacy denotes personal belief in one’s own ability to actively and positively face the ethical issues that may arise in the workplace, to overcome obstacles, and to develop and implement ethical solutions to ethical dilemmas (Hannah and Avolio, 2010; May et al., 2014). Prior research argues that the stronger the moral self-efficacy, the more perseverant people are in their self-controlling efforts and the greater is their resistance to unethical behavior (Wang et al., 2013). Therefore, we assume that moral self-efficacy would buff the relationship between political acts and interpersonal deviance. *Accordingly, the second research question leading this study is: Are the effects of political acts on interpersonal deviance contingent on their job strain and moral efficacy?*

In addition, job strain and moral self-efficacy are not independent. Research suggests that job strain serves as detrimental feedback to poor proficiency in the work domain (Dahling et al., 2013). In the long run, job strain can lead to more enduring negative outcomes, such as emotional exhaustion, burnout, and depression (Schmitt et al., 2016). Job strain is a barrier to the extent that it creates pressure and erodes employees’ confidence in behaving appropriately according to ethical rules within the organizations (Kavanagh and Bower, 1985). Based on the aforementioned arguments, this study examines how job strain moderates the relationship between daily political acts and interpersonal deviance. Specifically, the mediating role of moral efficacy is analyzed in order to answer the third question of this study: *Does moral efficacy mediate the moderating effect of job strain on the relationship between daily political acts and interpersonal deviance?*

**FIGURE 1:**
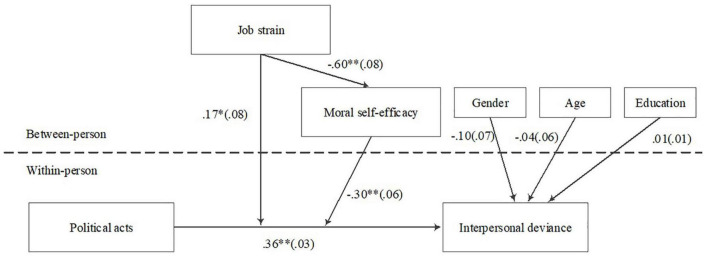
Conceptual model and results of structural equation model. *N* = 760 at the within-person level; *N* = 152 at the between person level. *p < 0.05; ^**^p < 0.01.

Drawing on the literature of political acts, interpersonal deviance, and ego depletion theory, a theoretical model was developed (see Figure 1). To test the conceptual model, the experience sampling method was adopted for when and how daily political acts induced interpersonal deviance. The research provides several contributions toward expanding political act and ego depletion. Firstly, by focusing on political actors, we explore the proximal relationship between political acts and interpersonal deviance, extending the outcome of political acts. Secondly, our research further explores the boundary condition under which political acts are more or less likely to be transformed into interpersonal deviance by examining the moderating role of job strain and moral efficacy. Thirdly, this research expands the ego depletion theory by presenting a theoretical explanation for the amplifying role of job strain in the ego depletion process through examination of the mediating role of moral efficacy.

## Literature Review and Hypothesis Development

### Political Acts and Interpersonal Deviance

Political acts are socially influential actions that aim to protect and promote an individual’s self-interests (Cheng et al., 2022). Such acts have a double-edged effect on the actors themselves. On the one hand, political acts are adopted as coping strategies to retain a sense of control when an individual is confronted with hindrance stressors. On the other hand, political acts sustainably consume actors’ self-regulation resources; this exacerbates ego depletion and undermines task performance (Sun and Chen, 2017).

Time is a valuable resource in the workplace. It is fixed in length and divided into several segments to complete anticipated tasks (Koopman et al., 2016). Although political acts are performed to protect individuals’ self-interests by cultivating resources from coworkers, they impede work progress by distracting actors from routine work behavior. Impeded work progress requires actors to invest more self-regulatory resources to facilitate the completion of tasks, resulting in enhanced ego depletion (Xia et al., 2020). In addition to impeded work progress, political acts require actors to engage in emotional labor and to invest time, energy, and attention (Ferris et al., 2002). Sun and Chen (2017) pointed out that political behavior consumes a finite pool of self-regulatory resources, thereby enhancing ego depletion. Ego depletion has been associated with interpersonal deviance (Zhang et al., 2021). Political acts are self-serving in interactions with coworkers and cause changes in interpersonal relationships at work (Gabriel et al., 2018). Interpersonal deviance is sensitive to such changes (Wu et al., 2014). When undergoing ego depletion, actors lack sufficient self-control resources to focus on the work and are more inclined to engage in interpersonal deviance to retain control in their interactions with coworkers. Thus, this study hypothesizes as follows:

Hypothesis 1. Political acts are associated with interpersonal deviance on a daily basis.

### Moderating Role of Chronic Job Strain

Ego depletion theory posits that self-control resources are limited. These resources are occupied by the preliminary activity and impact downstream behavior (Lin et al., 2016). Whenever activities consume considerable self-control resources, individuals become more likely to perform unethical behaviors (e.g., act rudely or steal money from colleagues), which would help them to recover from an ego depletion state (Li et al., 2019).

Given that time at work is limited and fragmented, employees strive to finish work-related goals on time (Koopman et al., 2016). A political act is a self-serving behavior. Employees engage in political acts in an attempt to influence the perceptions and behavior of other members, such as by taking credit for others’ work and blaming others for mistakes (Gabriel et al., 2018). A political act is typically a non-work behavior, which occupies limited work time and self-control resources. For the time occupied by political acts, employees could better devote themselves to work, which would result in further loss of self-control resources (Koopman et al., 2016). Because employees are motivated to avoid further depletion of self-control resources, they are less likely to direct resources toward prohibiting interpersonal aggressive behavior, and opportunities for interpersonal deviance arise (Rosen et al., 2016). Thus, we theorize that political acts in the morning are positively correlated with interpersonal deviance in the evening.

Ego depletion literature also posits that individual characteristics moderate the depletion process of self-control resources (Gabriel et al., 2018). Job strain is a subjective reaction to an unpleasant experience evoked by stressful work events (Hurrell et al., 1998). Job strain may derive from exposure to unfavorable job stressors (e.g., high task demands and organizational politics) and threaten individuals’ self-control resources (Schmitt et al., 2016). Previous research has found that self-control resources, which are used for maintaining effective functioning and appropriate work behavior, are depleted under high job strain (Prem et al., 2016).

Although political acts are instrumental to obtaining more resources for actors, the resources provided by political acts are unlikely to be invested into inhibiting interpersonal deviance. More likely, they are available to be used for keeping up effective functioning, including coping with job strain (Schmitt et al., 2016). When experiencing high job strain, mobilization of self-control resources is required to ensure basic and effective functioning rather than investment into inhibition of interpersonal deviance. In contrast, the resource depletion process is less affected by job strain. Thus, we theorize that:

Hypothesis 2. Chronic job strain moderates the relationship between political acts and interpersonal deviance such that the relationship is stronger for high levels of job strain than for low levels of job strain.

### Moderating Role of Moral Self-Efficacy

Moral efficacy is also an important individual difference variable that has received increasing attention in recent research on ethical behavior (Lee et al., 2017). Moral efficacy reveals people’s confidence in their abilities to execute ethical decisions and to perform appropriately according to ethical rules (Owens et al., 2019). Moral self-efficacy can be regarded as a determinant of ethical behavioral intention, and those with high moral efficacy tend to view inhibiting ethical behavior as less difficult. Individuals with high moral self-efficacy can inhibit, rather than engage in, interpersonal deviance with fewer self-control resources (Wang et al., 2013).

As aforementioned, employees with high moral self-efficacy are more perseverant in their self-control capabilities and achieve greater success in resisting temptations to behave in ways that violate ethical rules. Therefore, we assume that under the condition of high moral efficacy, the positive relationship between political acts and interpersonal deviance will be weaker because moral self-efficacy drives actors to direct their attentional resources toward inhibition of interpersonal deviance, whereas under the condition of low moral self-efficacy, attentional resources are used to maintain effective functioning rather than to inhibit interpersonal deviance, thereby strengthening the relationship between political acts and interpersonal deviance. Thus, we theorize that:

Hypothesis 3. Moral self-efficacy moderates the relationship between political acts and interpersonal deviance such that the relationship is stronger for low levels of moral self-efficacy than for high levels of moral self-efficacy.

### Mediated Moderation Model

We assume that chronic job strain and moral self-efficacy moderate the relationship between political acts and interpersonal deviance. However, chronic job strain and self-efficacy are inter-related. Job strain derives from an interaction of employees’ personal resources and their job demands (Schmitt et al., 2016). When job demands exceed employees’ abilities, skills, and resources, the unpleasant subjective experience of job strain may arise (Dicke et al., 2018). Thus, job strain is an indicator of poor performance proficiency in the work domain. Increased job strain drives pressure to perform according to organizations’ ethical rules and to inhibit employees from investing resources toward developing the confidence they need to perform ethically (Reisel et al., 2010). In this vein, we assume that job strain is negatively correlated with moral efficacy.

We suggest that chronic strain can decrease moral efficacy and moderate the relationship between political acts and interpersonal deviance on a daily basis. The unfavorable subjective experience of job strain may internalize their hardship as evidence of a failure experience and detract from moral efficacy (Hobman et al., 2009). Such a disadvantage facilitates ego depletion, thereby enhancing the relationship between political acts and interpersonal deviance. Therefore, we suggest that interpersonal deviance is a response to the depletion of attentional or self-control resources caused by political acts, but that it is contingent on employees’ job strain, which decreases moral efficacy, which in turn buffers the depletion process. Hence, we hypothesize the following:

Hypothesis 4. Moral self-efficacy mediates the moderating role of chronic job strain on the relationship between political acts and interpersonal deviance.

## Materials and Methods

### Participants

According to prior studies concerning interpersonal deviance and political acts, we have set several criteria for the sample select: (1) working full-time; (2) working from office rather than from home; (3) living and working in mainland, China. We contacted 3 MPA (Master of Public Administration) students in a university in Jiangxi Province, China, for help. They were working as human resource managers in their organizations and helped us to recruit a subject pool of 200 workers. We explained to the participants our research purpose, detailed research procedure and rewards, and 161 workers confirmed their participation in our survey. The sampling procedures were approved by the Ethics Committee and were in line with the 1964 Helsinki Declaration. Informed consent was signed and obtained from all the respondents in this study. We formed a research group on WeChat, a universal social media platform in China, and invited all the participants to join the research group. The research assistants sent the links to the questionnaires to the research group at the specified time. They were also in charge of identifying respondents who did not complete the questionnaire within the specified time. All the questionnaires were completed *via* mobile phone, and the respondents received 25¥ RMB (≈ 3.94 USD).

The survey was conducted in October 2019 and consisted of two stages. In the first stage, on a Sunday, participants were asked to finish a baseline survey, including gender, education, age, chronic job strain, and moral self-efficacy. During the second stage, they were asked to complete a midday (from 11:00 to 13:00) questionnaire assessing political acts and an evening (from 18:00 to 20:00) questionnaire assessing interpersonal deviance. In each questionnaire survey, the respondents were required to write down a special code, formed by the abbreviation of their Chinese names and the last two numbers of their mobile phone number. We used the code and the date automatically recorded by the websites to match the data.

Finally, 760 cases nested in 152 samples were returned for analysis, with 9 participants failing to finish the entire research process. The research yielded an effective response rate of 94.4%. The percentage of male samples was 53.9%, and the average age of the respondents was 26.84 (±3.27) years. With regard to education, 11.8% of the samples had a college certificate or below, 61.8% had a bachelor degree, and 26.3% had a master’s degree or above.

### Measures

As the scales used in this study were originally presented in English peer-review journals, we translated them into Chinese following a rigorous back translation procedure (Brislin, 1983). A five-point Likert scale was adopted to represent the variables, ranging from 1 with “strongly disagree” to 5 with “strongly agree.”

#### Daily Measures

##### Political Acts

Three items were used from the scale developed by Hochwarter et al. (2003). The items were, “Today, I engaged in self-serving behavior,” “Today, I spent time winning the approval of supervisors or managers who can help me,” “Done what is best for me, not what is best for the organization.” The average Cronbach’s alpha was 0.90, and the McDonald’s Omega was 0.89.

##### Interpersonal Deviance

Three items were adapted from the scale developed by Bennett and Robinson (2000). The items were, “Today, I made fun of someone at work,” “Today, I said something hurtful to someone at work,” “Today, I played a mean prank on someone at work.” The scale yielded a Cronbach’s alpha of 0.91 and a McDonald’s Omega of 0.92.

#### Baseline Survey

##### Job Strain

Three items were used from the job-induced tension scale developed by Motowidlo et al. (1986). The items were, “I feel a great deal of stress because of my job,” “My job is extremely stressful,” “Very few stressful things happen to me at work (R).” The Cronbach’s alpha of the scale was 0.72 and the McDonald’s Omega was 0.71.

##### Moral Self-Efficacy

It was measured by three items used by Owens et al. (2019). Items were, “I was self-assured about my capabilities to perform my work activities in an ethical manner,” “I am confident about my ability to do my job in a way that meets the organization’s ethical standards,” “I have mastered the ethical rules, regulations and skill necessary for my job.” The scale yielded a Cronbach’s alpha of 0.96 and a McDonald’s Omega of 0.96.

##### Control Variables

The demographic variables of gender, education, and age were used as control variables for the predictors of interpersonal deviance (Miller, 2015; Itzkovich and Heilbrunn, 2016; Wu et al., 2020).

### Analytic Strategy

The data at the between-personal level (e.g., gender, education, age, job strain, and moral self-efficacy) and at the within-personal level (e.g., political acts and interpersonal deviance) were collected through experience sampling method, representing the nested data structure. The multilevel data modeling methods including hierarchical linear modelling (HLM) and multilevel structural equation modelling (MSEM) were adopted in this study.

First, the within-personal variance in the daily variables were examined using HLM. The results indicated that 66% variance for political acts and 88% variance for interpersonal deviance at the within-personal level, justifying the application of the multilevel analysis. Second, we used HLM to analyze the data to calculate means and standard deviations in this study. Third, the hypotheses testing was conducted using HLM to test the direct effects and moderation effects, and using MSEM to run the moderated mediation analysis with random slope and robust estimators to indicate mediation effects and moderated mediation effects.

## Results

### Descriptive Statistics and Multilevel Confirmatory Factor Analysis

The means, standard deviations, and correlation coefficients of the focal variables in both within- and between-person levels are shown in Table 1. We found a positive relationship between political acts and interpersonal deviance (*r* = 0.34, *p* < 0.01) and a negative relationship between chronic job strain and moral self-efficacy (*r* = −0.41, *p* < 0.01).

**TABLE 1:** Means, standard division, and correlation analysis.

Within-person level (*N* = 760)	Mean	SD	Kurtosis	Skewness	1	2			
1. Political acts	3.71	0.83	−0.15	−0.39	(0.90)				
2. Interpersonal deviance	3.26	0.91	−0.38	−0.27	0.34**	(0.91)			

**Between-person level (*N* = 152)**					**1**	**2**	**3**	**4**	**5**

1. Gender									
2. Education					0.02				
3. Age	26.84	3.27			0.04	−0.10**			
4. Job strain	3.84	0.42	−0.33	−0.32	−0.01	−0.03	0.00	(0.72)	
5. Moral self-efficacy	3.27	0.64	−0.41	−0.31	−0.17**	0.05	0.22**	−0.41**	(0.96)

Values in the parenthesis are Cronbach’s Alpha. **p < 0.01.

We ran a multilevel confirmatory factor analysis for political acts and interpersonal deviance at the within-person level, and for chronic job strain and moral self-efficacy at the between-person level. The results indicated that the four-factor conceptual model fit the data well (χ^2^ = 23.23, df = 12, χ^2^/df = 1.94 < 3, RMSEA = 0.04 < 0.08, SRMR_Within_ = 0.03 < 0.08, CFI = 0.99 > 0.90, TLI = 0.99 > 0.90) (Sivo et al., 2006).

### Results of Multilevel Regression and Structural Equation Model

The hypotheses were tested through a multilevel moderation analysis and a multilevel structural equation model analysis using Mplus software (Version 7.0). Before we ran the hierarchical linear model, political acts, and interpersonal deviance were group centered, and continuous between-person variables (i.e., age, chronic job strain, and moral self-efficacy) were grand centered.

The results of the analysis were shown in Table 2. Both the results in Model 1 (γ = 0.37, *p* < 0.01) and Model 2 (γ = 0.36 *p* < 0.01) indicated the positive association between political acts and interpersonal deviance. Hypothesis 1 was supported. The result of model 1 indicated that the interactive item of political acts with chronic job strain was significantly associated with interpersonal deviance (γ = 0.35, *p* < 0.01). To further test the moderating role of job strain, we adopted the Monte Carlo bootstrapping test in order to calculate the confidence interval for the interactive effect.

**TABLE 2:** Results of hierarchical linear model.

Variables	Interpersonal deviance			
	Model 1		Model 2	
	γ	*SE*	γ	*SE*
Intercepts	3.62	0.19	3.48	0.15
Between-person level				
Gender	−0.23**	0.08	0.15	0.07
Education	–0.01	0.07	–0.04	0.05
Age	0.03	0.01	0.01	0.01
Chronic job strain	−0.44**	0.08		
Moral self-efficacy			0.65**	0.05
Within-person level				
Political acts	0.37**	0.04	0.36**	0.03
Interactive item				
Political acts × Chronic job strain	0.35**	0.07		
Political acts × Moral self-efficacy			−0.36**	0.05
Pseudo *R*2	0.21		0.41	

760 cases nested in 152 samples. ** p < 0.01.

**FIGURE 2:**
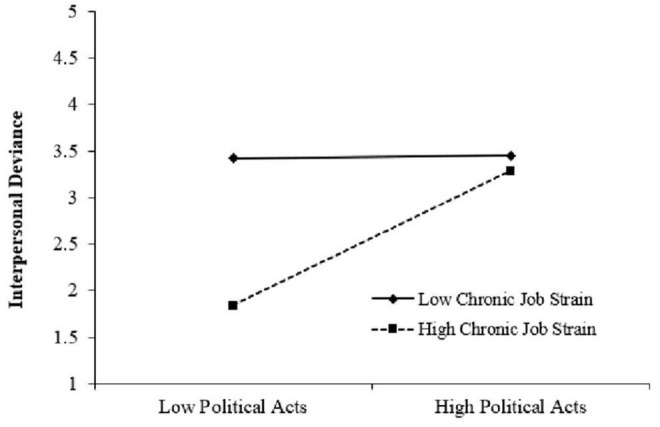
Moderating effect of Chronic Job Strain.

The results in Table 3 showed that the relationship between political acts and interpersonal deviance was significant under the condition of low job strain (Effect = 0.22, 95% CI = [0.13, 0.32]). However, under the condition of high job strain, the relationship was stronger (Effect = 0.52, 95% CI = [0.42, 0.62]). The slope difference was also significant (Effect = 0.30, 95% CI = [0.18, 0.41]), justifying the moderating role of job strain, which was shown in Figure 2. Hypothesis 2 was supported.

**TABLE 3:** Results of Monte Carlo bootstrapping test.

Moderating role of chronic job strain	Mean	*SD*	95% LLCI	95% ULCI
Low chronic job strain (M − SD)	0.22	0.05	0.13	0.32
High chronic job strain (M + SD)	0.52	0.05	0.42	0.62
Difference	0.30	0.06	0.18	0.41
**Moderating role of moral self-efficacy**				
Low moral self-efficacy (M − SD)	0.58	0.04	0.50	0.67
High moral self-efficacy (M + SD)	0.14	0.04	0.05	0.22
Difference	−0.45	0.06	−0.57	−0.32
**Mediated moderation model**				
Indirect effect (Moderating effect of chronic job strain through moral self-efficacy)	0.18	0.04	0.10	0.27
Direct effect (Moderating effect of chronic job strain)	0.17	0.08	0.02	0.32

*Bootstrapping = 20,000.*

**FIGURE 3:**
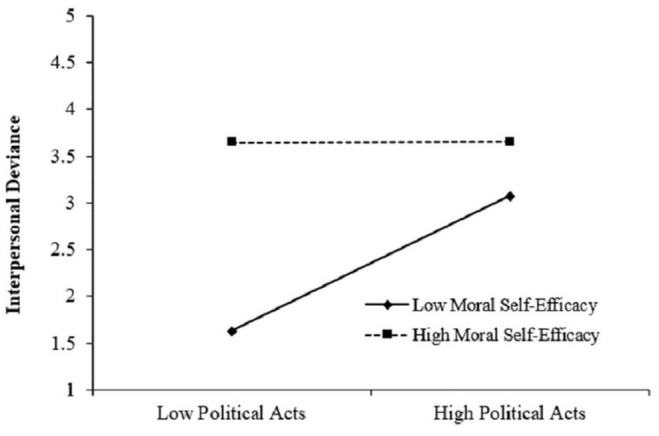
Moderating effect of moral self-efficacy.

The interactive item of political acts with moral self-efficacy was significantly correlated with interpersonal deviance (γ = −0.36, *p* < 0.01). In order to further test the moderating role of moral self-efficacy, we ran the Monte Carlo bootstrapping test to calculate the confidence interval for the interactive effect. The results in Table 3 showed that the relationship between political acts and interpersonal deviance was significant under the condition of low moral self-efficacy (Effect = 0.58, 95% CI = [0.50, 0.67]). However, under the condition of high moral self-efficacy, the relationship was weaker (Effect = 0.14, 95% CI = [0.05, 0.22]). The slope difference was also significant (Effect = −0.45, 95% CI = [−0.57, −0.32]), justifying the moderating role of moral self-efficacy, which was shown in Figure 3. Hypothesis 3 was supported.

To holistically test the mediated moderation model, we follow the suggestion from Roesch et al. (2010) to run a multilevel structural equation model. The results in Figure 1 indicated that the relationship between job strain and moral self-efficacy was significant (γ = −0.60, *p* < 0.01). The moderating effect of moral self-efficacy was significant (γ = −0.30, *p* < 0.01), and the moderating effect of chronic job strain was also significant (γ = 0.17, *p* < 0.05). Furthermore, we ran the Monte Carlo bootstrapping test on the mediated moderation model. The indirect effect was significant (Effect = 0.18, 95% CI = [0.10, 0.27]), while the direct effect was insignificant (Effect = 0.17, 95% CI = [0.02, 0.32]). Hypothesis 4 was supported.

## Discussion

Through the experience sampling method, we focused on the relationship between political acts in the morning and interpersonal deviance in the evening. We explored the boundary condition under which political act is more or less likely to be transferred into interpersonal deviance by examining the amplifying role of chronic job strain and the buffering role of moral self-efficacy. Moreover, this study built a mediated moderation model to demonstrate why chronic job strain can be regarded as a moderator. As expected, the moderating role of chronic job strain was mediated by moral self-efficacy. By addressing the relationship between political acts and interpersonal deviance, this study makes three contributions to the literature.

First, the current research provides a possible explanation for the relationship between political acts and interpersonal deviance. A political act is instrumental and self-serving, which may potentially obtain resources for actors from their colleagues and even leaders (Gabriel et al., 2018). However, it is also a non-work behavior, which impedes work progress and occupies work-time, resulting in depletion of self-control resources (Sjåstad and Baumeister, 2018). Importantly, we note that prior research theoretically addresses the negative implications for political acts on the organizational level (Crawford et al., 2019). This research expands on ego depletion theory and finds that political acts have negative implications for interpersonal relationships in terms of increased interpersonal deviance. Our research extends the proximal outcomes of political acts, enriching the political act literature.

Second, this study offers a theoretical boundary for the positive relationship between political acts and interpersonal deviance that contributes to political acts. Most previous studies have regarded the political act as a kind of resource-gaining behavior and found that it may contribute to self-interests, such as leaders’ recognition and appraisal (Gabriel et al., 2018). Our findings reveal that the political act has a stronger relationship with interpersonal deviance in the case of high chronic job strain and low moral efficacy. Although the political act is instrumental, its gained resource can only be used to cope with ego depletion when job strain is low and moral efficacy is high. Employees are required to use job resources to ensure effective functioning in order to cope with job strain, leaving limited self-control resources to inhibit interpersonal deviance (Schmitt et al., 2016). In contrast, moral self-efficacy enhances employees’ confidence to handle ego depletion, and drives them to invest self-control resources to prevent interpersonal deviance (Rathert et al., 2016). The present study contributes to political act literature by providing a more comprehensive view of how employees experiencing different levels of job strain and moral self-efficacy respond to ego depletion caused by political acts.

Third, although our primary contribution is to political act research, this research also extends our understanding of the ego depletion theory by examining the moderating role of chronic job strain *via* moral self-efficacy. Prior ego depletion theory studies have only examined the amplifying role of chronic job strain and buffering role of moral self-efficacy in the ego depletion process separately (Parker et al., 2017; Tuzun et al., 2017). However, scarce research tries to simultaneously examine the different moderating effects of chronic strain and moral self-efficacy, and the underlying mechanism in which these two individual characteristics influence each other. As consistent with prior studies, this study found that chronic job strain would strengthen while moral self-efficacy would attenuate the ego depletion process caused by political act (Diestel and Schmidt, 2011; Park and Sprung, 2015). Furthermore, the study attempts to explain the moderating role of chronic job strain according to its shaping role in moral efficacy. This study finds that chronic job strain will first detract from employees’ moral efficacy, then influence the relationship between political acts and interpersonal deviance. By doing so, this study explains the underlying mechanism through which individual characteristics impact the ego depletion process, extending the scope of the ego depletion theory.

It should be noted that there was a nonsignificant relationship between ego depletion effects and task performance, implying a need to reconsider the application of ego depletion theory in psychological research. Recent studies have mainly adopted ego depletion theory to explain the emergence of unethical behavior when confronted with unfavorable situations, such as abusive supervision, work incivility, and hindrance stressors (Rosen et al., 2016; Mackey et al., 2020; Xia et al., 2020). Extending this line of research, recent studies have begun to explore the effects of ego depletion on unethical behavior from an actor-centric perspective. Their research purpose is to explain how altruistic behavior turns into unethical behavior through the process of ego depletion (Lin et al., 2016; Qian et al., 2020). Enlarging the scope of this line of research, the present study explores the circumstances under which political acts, an unethical self-serving behavior, are transformed into interpersonal deviance, an unethical harmful behavior, at work. Political acts demand an investment of time, emotional resources, and attention, resulting in ego depletion and leading to interpersonal deviance. Moreover, this study highlights the fact that this process is shaped by chronic job strain and moral self-efficacy. Our study provides a new research paradigm for the application of ego depletion theory and points to a direction for future research.

### Practical Implications

Our findings provide insights into how organizations might inhibit the negative influences of political acts without inducing interpersonal deviance. To inhibit the negative outcomes of political acts, managers should consistently decrease chronic job strain. Research has provided evidence for the influences of effective intervention (e.g., mindfulness-based intervention, employee assistance programs, and job crafting interventions) to combat job strain (Caillier, 2016; Rudolph et al., 2017; Bostock et al., 2019). Managers should adopt related intervention programs to inhibit political acts from being transferred into interpersonal deviance, which exerts greater detrimental effects on team effectiveness.

Furthermore, moral self-efficacy should be taken into consideration. Ethical leadership is a critical antecedent to enhancing employees’ moral efficacy (Lee et al., 2017). Thus, supervisors should strive to provide ethical leadership in order to nurture their employees’ moral efficacy, which would help employees to regulate behavior and cope with ego depletion.

### Limitations and Future Research

Our research has several limitations indicating orientations for future research. First, our research attempts to explore the episodic relationship between political acts and interpersonal deviance. Thus, we adopted the experience sampling method to collect daily data and found a positive association between the two variables. However, we cannot establish a firm causal relationship because we did not manipulate political acts. For future research, a cross lagged panel design and experiment design are recommended to further verify the causal influence of political acts on interpersonal deviance (Loiselle, 2018; Gagné et al., 2019).

Second, we cannot rule out common method variance (CMV; Podsakoff et al., 2003). To control the CMV, we collected data *via* a two-wave experience sampling method in the midday and evening, respectively, over five consecutive days. However, the data were collected *via* self-reported questionnaires. Although the results of multilevel confirmatory analysis suggested that CMV was not a serious concern in the present study, it would be better for future research to use multi-sourced data in order to avoid CMV. For example, interpersonal deviance can be evaluated by colleagues.

Third, our samples are Chinese full-time workers in public sectors, which limits the external validity of our results. Culture plays a vital role in shaping political acts. Traditional Chinese collectivism culture discourages political acts, which may induce greater ego depletion for actors in Chinese firms (Moorman and Blakely, 1995). Future research should take culture-related variables into consideration in order to test the robustness of our research.

## Data Availability Statement

The raw data supporting the conclusions of this article will be made available by the authors, without undue reservation.

## Ethics Statement

The studies involving human participants were reviewed and approved by the Ethics Committee of University of Chinese Academy of Social Sciences and were in line with the 1964 Helsinki Declaration and its later amendments or comparable ethical standards. The patients/participants provided their written informed consent to participate in this study.

## Author Contributions

JX: supervision and funding acquisition. JZ: writing–review and editing. ZL: supervision. ZZ: conceptualization, methodology, formal analysis, and writing–original draft. All authors contributed to the article and approved the submitted version.

## Conflict of Interest

The authors declare that the research was conducted in the absence of any commercial or financial relationships that could be construed as a potential conflict of interest.

## Publisher’s Note

All claims expressed in this article are solely those of the authors and do not necessarily represent those of their affiliated organizations, or those of the publisher, the editors and the reviewers. Any product that may be evaluated in this article, or claim that may be made by its manufacturer, is not guaranteed or endorsed by the publisher.
